# Complete mitochondrial genome of Antarctic krill *Euphausia superba* (Eucarida: Euphausiacea)

**DOI:** 10.1080/23802359.2017.1413306

**Published:** 2017-12-08

**Authors:** Ming Zhao, Mengdi Zhao, Chunyan Ma, Chunlei Feng, Luming Wang, Lingbo Ma

**Affiliations:** Key Laboratory of East China Sea & Oceanic Fishery Resources Exploitation and Utilization, Ministry of Agriculture, East China Sea Fisheries Research Institute, Chinese Academy of Fishery Sciences, Shanghai, China

**Keywords:** *Euphausia superba*, mitochondrial genome, control region, ND2, molecular maker

## Abstract

Antarctic krill *Euphausia superba* is a very important species in Antarctic ecosystem. The mitochondrial genome of *E. superba* was completed with 16,591 bp in length, gene arrangement and order was the same as previous studies. The overall A + T content is 68.91% and the control region A + T content is 78.17%. Alignment with other two partial mitochondrial genome revealed that ND2 region possessed many unusual variation sites. The phylogenetic tree showed that *E. superba* clustered with other two species of Euphausiacea. This study filled in the gap of the krill mitogenome and suggested putative markers for population study.

Selective pressure for different regions of mitochondrial genome was different. *Euphausia superba* was the most important species in Antarctic region and some efforts have been tried to obtain the complete mitogenome of krill but failed (Machida et al. 2004; Shen et al. 2010; Johansson et al. 2012). Antarctic krill was sampled from 63°6′S, 58°45′W (S63W58). Genomic DNA of one individual with morphological identification was extracted using TIANamp Marine Animal DNA Kit (TIANGEN Biotech, Beijing, China) following the manual. The mitochondrial genome DNA of *E. superba* was a circular molecular of 16,591 bp in length with a predicted control region of 1550 bp in length. Gene arrangement and order were the same as described by Shen et al. (2010). The overall A + T content was 68.91% for the H-strand and the control region has the highest A + T content (78.17%), while COIII has the lowest (62.55%).

The amino acids sequences of ND2 from three mitochondrial genomes of *E. superba* were predicted and aligned using the DNAMAN software. ND2 amino acids sequences of *E. superba* (S63W58) and *E. superba* (WS) was complete same but sixteen amino variations were found between *E. superba* (S63W58) and *E. superba* (PB) which might be too plentiful in one species (Figures 1).

**Figure 1.**
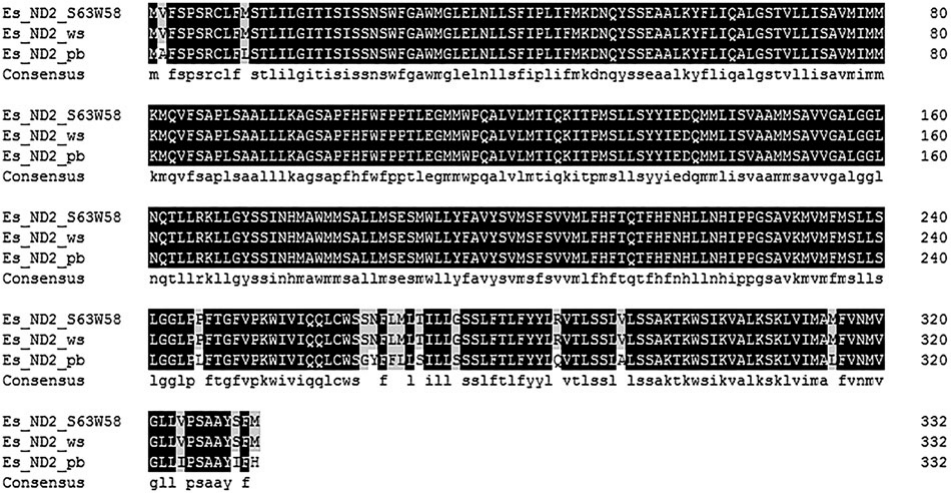
Alignment of ND2 amino acids sequence of three mitochondrial genome.

Phylogenetic tree was constructed based on the 12 protein coding genes except for ND6 used MEGA7 with the maximum-likelihood method (MEGA Inc., Tampa, FL). It revealed that *E. superba* (S63W58) first clustered with other two *E. superba*, and *E. superba* has the closest relationship with *Euphausia pacifica*. All three species of Euphausiacea clustered to form a big branch (Figure 2).

**Figure 2.**
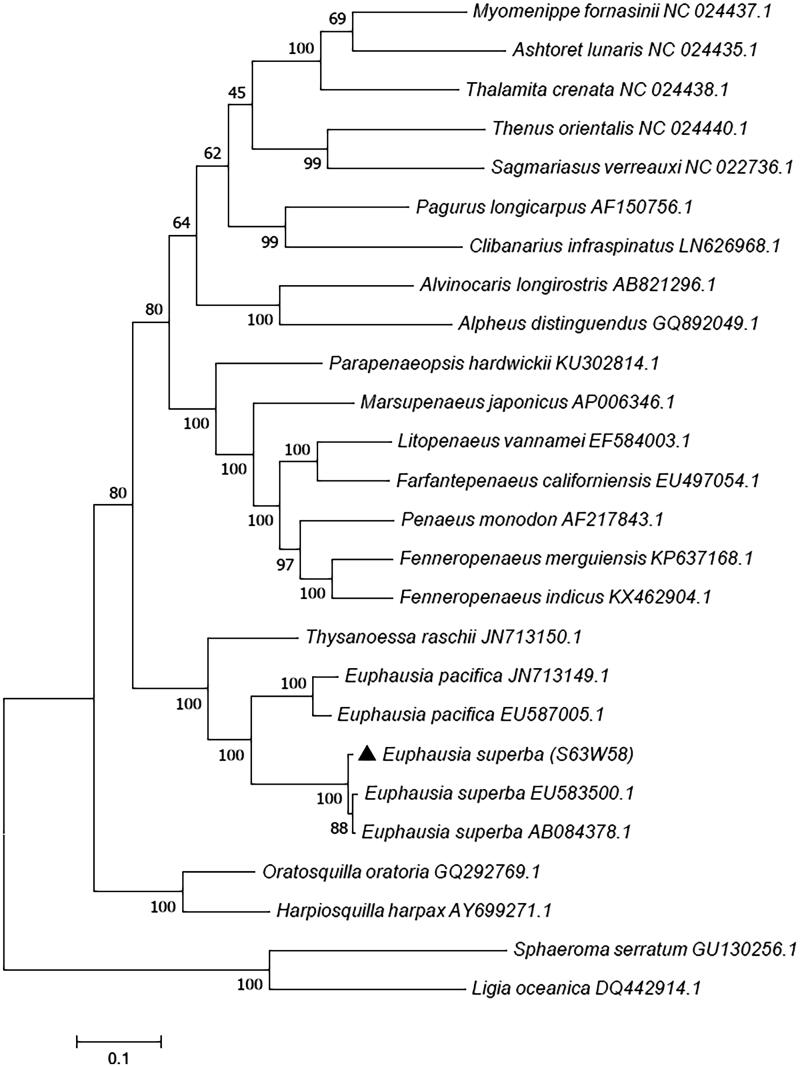
Phylogenetic tree of *Euphausia superba* based on 13 protein-coding genes using maximum-likelihood method.
